# Human health impact assessment and temporal distribution of trace elements in Cop**ș**a Mică- Romania

**DOI:** 10.1038/s41598-021-86488-5

**Published:** 2021-03-29

**Authors:** Katalin Bodor, Zsolt Bodor, Alexandru Szép, Róbert Szép

**Affiliations:** 1 Department of Bioengineering, Socio-Human Sciences and Engineering, Faculty of Economics, Sapientia Hungarian University of Transylvania, Libertăţii Sq.1, 530104 Miercurea Ciuc, Romania; 2 Doctoral School of Chemistry, Faculty of Natural Sciences, University of Pécs, Ifjúság 6, Pécs, 7624 Hungary; 3 Institute for Research and Development for Hunting and Mountain Resources, Str. Progresului 35B, 530240 Miercurea Ciuc, Romania; 4 Department of Food Engineering, Faculty of Economics, Socio-Human Sciences and Engineering, Sapientia Hungarian University of Transylvania, Libertăţii Sq.1, 530104 Miercurea Ciuc, Romania

**Keywords:** Environmental sciences, Risk factors

## Abstract

The present study aims to analyze the temporal variations of PM_10_ and to assess the health risk indexes caused by trace elements from particulate matter (PM_10_) via inhalation, ingestion, and dermal absorption by adults and children in Copșa Mică (Romania) during 2009–2019. The results revealed a high multi-annual mean concentration of PM_10_ and trace elements. The analyzed air pollutants showed a decreasing trend during the studied years, therefore 44.11%, 43.48%, 36.07%, 16.02%, and 15.80% lower values were observed for As, Cd, Ni, PM_10_, and Pb, respectively, due to environmental regulations. The daily exceedance percentage of Pb and Cd was very high, representing 21.74% and 11.26%, followed by PM_10_ and As concentrations with 4.72% and 3.92%. The ratio between the trace element concentration measured in Copșa Mică and the country average was 2.46, 4.01, 2.44 and 10.52 times higher for As, Cd, Ni and Pb. The calculated Hazard Quotient values via inhalation were higher than the safe limit (1), which accounted 1.81, 3.89 and 4.52, for As, Cd and Ni, respectively, indicating that the trace elements might present a non-carcinogenic risk to both adults and children. Furthermore, the concentration of all studied trace elements in Copșa Mică showed cancer risk for adults via inhalation and dermal absorption as well.

## Introduction

Particulate matter (PM_10_) is an important air pollutant consisting of small particles with an aerodynamic diameter less than or equal to a nominal 10 µm having a significant impact on human health, causing serious disease (respiratory, cardiovascular) and premature death worldwide^1–3^. The particulate matters originated from different (industrial, traffic) emissions may contain several toxic trace elements, such as As, Cd, Cr, Cu, Zn, Pb and Ni. These toxic elements may enter the body via three different entry routes: ingestion, inhalation and skin absorption^4^. Many studies have been published on the topic of inhalable particles associated with trace elements that have an increased effect on lung and cardiopulmonary morbidity and mortality^5,6^. The annual acceptable limit for the most important air pollutants was established in the Air Quality Standards including PM_10_ (20 µg m^-3^), Pb (0.5 µg m^-3^), As (6 ng m^-3^), Cd (5 ng m^-3^) and Ni (20 ng m^-3^) as well^7^.

The trace elements concentration is strongly associated with the source types^8^, hence the most common source of As, Cd and Pb, are coal combustion^9^ and metal smelting industry, which are also responsible for the emission of trace elements^10,11^. The studied town Copșa Mică was best known as one of the most polluted cities in Europe where the carbon black, lead and zinc production industry heavily polluted the ecosystems. Due to this pollution, in Copşa Mică the life expectancy is with 9 years below the Romanian average, where the spread of lung cancer, lead poisoning, bronchitis, rickets, finger-twitching, learning difficulties, impotence, asthma, stunted growth, depression and alcoholism are very common morbidities^12,13^. Furthermore, the temporal variations of PMs and trace elements highly depend on the emission sources and specific meteorological conditions^14,15^.

The trace elements concentration of particulate matter measured near industrial areas has been studied and reported worldwide, in the USA by Landis et al.^16^ and Xia and Gao^5^, in Korea by Kim et al.^17^, in Greece by Manalis et al.^18^, in Italy by Mazzei et al.^19^, Prodi et al.^20^ and Amodio et al.^21^, in Turkey by Cetin et al.^22^, in Spain by Querol^23^, in China by Zhang et al.^24^ Tian et al.^25^ and Du et al.^4^, in Taiwan by Jiun-Horng et al.^26^, in Poland by Pastuszka et al.^27^, in France by Hleins et al.^28^, in Australia by Mohiuddin et al.^29^, in the United Kingdom by Taiwo et al.^30^, in Romania by Dunea et al.^6^ and Proorocu et al.^31^. According to the findings reported, the level of trace elements near industrial areas can reach values which may represent serious health risks at local and regional levels. Therefore, it is mandatory to decipher the effects on human health, and in many cases the complex interactions are yet to be discovered.

The main objective of this research study was to analyze the heavy metals (As, Cd, Ni, Pb) temporal distribution determined from the PM_10_ and to assess the human health effect using the Hazard Quotient (HQ) and cancer risk (CR) methods, where three exposure pathways were considered: inhalation, ingestion and dermal absorption.

## Study area

### Sampling site

Copșa Mică (46°6′45″N 24°13′50″E) is a small industrial town located in Sibiu County, Romania, and was the most polluted city in Europe in 1991 due to heavy emissions from zinc, cadmium and lead refineries and carbon black plants. According to the literature, the uncontrolled emission over the years had a negative effect on air quality, soil, water, plant, animal and humans as well^32^. As a consequence of the uncontrolled air and soil pollution in recent decades has a demonstrable effect, since the trace elements were detected in the food chain, namely in honey^33^.

## Materials and methods

In the present study, the temporal variations of daily PM_10_ concentration and associated trace elements As, Cd, Ni, Pb were examined from January 2009 to August 2019. The raw data regarding daily pollutants concentrations were obtained from the National Air Quality Monitoring Network (www.calitateaer.ro*).* Therefore, during the studied period 3049 PM_10_, 2272 As, 2300 Cd, 2270 Ni and 2272 daily Pb sample was analyzed. Romania's climate is temperate continental transitioning, with four distinct seasons. In order to evaluate the seasonality aspect of pollution, the concentrations were grouped into four groups as follows; spring (March, April and May), summer—warm period (June, July and August), autumn (September, October and November) and winter—cold period (December, January and February).

The industrial monitoring station RO-SB-3(RO0186A) is situated in Copșa Mică with coordinate: latitude 46.11°N and longitude: 24.23°E, and 286 m asl. Trace elements were determined from the PM_10_ particulate matter fraction according to the SR EN 14,902 ”Ambient air quality from the fraction of PM_10_ particulate matter “ reference method. As described in this method, the collected particulate matter via aspiration was pre-treated in a microwave oven in a closed vessel, using concentrate nitric acid and hydrogen peroxide (30%). From the obtained solution, the trace element concentrations were measured with inductively coupled plasma mass spectrometry (ICP-MS).

### Statistical analysis

In order to decipher the temporal differences, descriptive statistics, monthly and annual trends were calculated, and the results were presented using box-plot diagrams. The study uses the 25th percentile of data as background levels. The box-plot used the median, the Q1 and Q3 quartiles, and the minimum and maximum data points to convey the level, spread, and symmetry of a distribution of data values^34^.

Using R (R 3.6.2) statistical program, the Spearman correlation analysis was carried to understand the relationship between the monthly mean pollutants (As, Cd, Ni, Pb, PM_10_) concentration and meteorological parameters (precipitation quantity, temperature, relative humidity, wind speed). The hierarchical cluster analysis method (Centroid Linkage, Correlation Coefficient Distance) was used to classify the PM_10_ and the trace elements (As, Cd, Ni, Pb) in into groups or clusters based on their similarities. The results were analyzed with Minitab 17.3.1 statistical software and the outcomes are presented in a dendrogram, while for the Principal Component Analysis the IBM SPSS Statistics 22. was applied.

### The health risk posed by heavy metals in PM_10_

According to the United States Environmental Protection Agency (USA-EPA), the human health effect caused by the heavy metals from PM_10_ was calculated via ingestion (*CDI*—chemical daily intake), inhalation (*EC*—exposure concentration), and dermal absorption (*DAD*—dermal absorption dose (EPA) for both groups children (ch) and adults (ad)^35^. Regarding the concentration of the pollutant, the multiannual mean value was used throughout the entire studied period (2009–2019).

1 $$CDI_{ing} = \left( {C \times IngR \times EF \times ED \times CF} \right)/\left( {BW \times AT} \right)$$

2 $$EC_{inh} = \left( {C \times ET \times EF \times ED} \right)/AT$$

3 $$DAD_{derm} = \left( {C \times SA \times AF \times EV \times ABS \times EF \times ED \times CF} \right)/\left( {BW \times AT} \right)$$

where *C*—is the metal multiannual concentration in PM_10,_ (μg m^-3^); *IngR—*Ingestion rate—(ch.:250 mg day^-1^, ad.:100 mg day^-1^); *EF*—Exposure frequency, (days year^−1^); *ED*—Exposure duration, (ch: 6 years, ad: 24 years); *CF*—Conversion factor, (10^−6^ kg mg^−1^); *BW*—Average body weight, (ch:15 kg, ad:70 kg); *AT*_*nc*_—Averaging time non-carcinogen, (ch:2190 day, ad: 8760 day); *ET*—Exposure time, (24 h day^−1^); *AT*_*c*_—Averaging time carcinogen; (ch:2190 day, ad: 25,550 day); *SA*—the skin surface area that contacts with the PM, (ch:2800 cm^2^, ad:3300 cm^2^); *AF*—Skin adherence factor for the airborne particulates, (0.2 mg cm^−2^); *ABS*—Dermal absorption factor (As: 0.03, Cd& other: 0.01); *ET*—Exposure time, (24 h day^−1^); *AT*_*nc*_—Average time for non-carcinogens, (ch: 52,560 h, ad: 210,240 h); *AT*_*c*_—Average time for carcinogens, (ad: 613,200 h).

HQ and CR caused by heavy metals in PM_10_ via ingestion, inhalation, and dermal contact was calculated using the following equations^35^. All of the parameters used in the calculation procedure were calculated according to EPA (2004).

4 $$HQ_{ing} = CDI/RfDo$$

5 $$HQ_{inh} = EC/\left( {RfCi \times 1000 \mu g mg^{ - 1} } \right)$$

6 $$HQ_{derm} = DAD/\left( {RfDo \times GIABS} \right)$$

7 $$CR_{ing} = CDI \times SFo$$

8 $$CR_{inh} = IUR \times EC$$

9 $$CR_{derm} = DAD \times \left( {SFo/GIABS} \right)$$

where *RfDo*—oral reference dose (mg kg ^-1^ day^-1^) (As: 3.00E−04, Cd: 1.00E−03, Ni: 1.10E−02, Pb: 3.50E−03); *RfCi*—inhalation reference concentrations (mg m^-3^) (As: 1.50E−05, Cd: 1.00E−05, Ni: 2.00E−05); *SFo*—oral slope factor (mg kg ^-1^ day^-1^) (As: 1.50E + 00, Pb: 2.80E−01); *GIABS*—gastrointestinal absorption factor (As&Pb:1, Cd: 0.025, Ni: 0.040); *IUR*—inhalation unit (µg m^-3^)^-1^ (As: 4.30E−03, Cd: 1.80E−03, Ni: 2.60E−04, Pb: 1.20E−05).

The trace elements (As, Cd, Ni, Pb), non-carcinogenic and carcinogen effect on human health was separately calculated for the children and adults, based on the different characteristics and exposure time. In order to assess the non-carcinogenic and carcinogenic risks the hazard quotient (HQ) and cancer risk (CR) approaches were used. For interpretation of HQ values: less than 1, no adverse health effects are expected as a result of exposure, while in the case of HQ greater than 1, then adverse health effects are possible. CR represents the increased probability of incident of tumor diseases above the general average due to the impact of carcinogenic compound's effects. The evaluation of CR for carcinogenic chemicals is taken into consideration and represents a risk, when the values varies from 10^−4^ to 10^−6^, representing that the cancer development during a human lifetime (70 years) is 1/10,000 or 1/1,000,000, respectively^35^. Values lower than 10^−6^ for individual chemicals and pathways show no cancer risks. Generally speaking, a cumulative cancer risk higher than 10^−4^ is not accepted, and the maximum tolerable value is 10^−5^.

## Results and discussions

### Statistical analysis

Through the statistical analysis the daily datasets were used. Within the studied period, the average concentration of PM_10_ in Copșa Mică was found to be 24.62 µg m^-3^, which is 23.1% higher than the WHO's acceptable limit (20 µg m^-3^)^7^. The average pollutant concentration between 2009 and 2019 in the studied area was 1.65 ng m^-3^ for As, 2.37 ng m^-3^ for Cd, 5.5 ng m^-3^ for Ni and 0.32 µg m^-3^ for Pb, respectively. During the studied period, the highest trace element concentration in PM_10_ was recorded for Pb, meanwhile the lowest one was observed in case of As (Table 1).

**Table 1:** Descriptive statistics of daily pollutants data and meteorological parameters in Copșa Mică.

	Ref.	Mean	N	Min	25P	Med	75P	Max	Stdev	CI95%	CV	% Exc
As, [ng m^−3^]	6.00	1.65	2272	0	0.61	1.00	1.74	27.25	2.07	1.56–1.73	1.26	3.92
Cd, [ng m^−3^]	5.00	2.37	2300	0.02	0.65	1.54	3.13	34.52	2.79	2.25–2.48	1.18	11.26
Ni, [ng m^−3^]	20.0	5.50	2270	0	1.44	4.09	8.99	62.68	4.82	5.31–5.70	0.88	0.79
Pb, [µg m^−3^]	0.50	0.32	2300	0	0.09	0.22	0.45	3.09	0.31	0.30–0.33	0.98	21.74
PM10, [µg m^−3^]	20	24.62	3049	0.24	12.72	21.8	32.7	118.1	14.87	24.09–25.15	0.6	4.72
Prec, [mm]	–	7.62	4062	0	0	2.2	12.8	112.2	11.78	7.26–7.98	1.55	–
T, [°C]	–	11.97	3461	0	4.73	11.49	19.05	28.78	7.71	11.71–12.23	0.64	–
RH, [%]	–	76.49	3461	34	70	77	85	100	11.57	76.11–76.88	0.15	–

*Ref.*—Annual acceptable limit; *Mean*—Average; *N*—Number of samples; *Min*—Minimum; *25P*—25th percentile; *Med*—Median; *75P*—75th percentile; *Max*—Maximum; *Stdev*—Standard deviation; *CI95%*—Confidence interval;* CV*—Coefficient of variation; *% Exc*—Percentage of exceedance;* Prec*—Precipitation quantity (annual); *T*—Temperature; *RH*—Relative humidity.

The results revealed that the exceedance percentage, based on the WHO Air Quality guideline, of Pb (> 0.5 µg m^-3^) and Cd (> 5 ng m^-3^) was very high. The daily permissible concentration was exceeded with 21.74% and 11.26%, respectively, followed by the PM_10_ (> 20 µg m^-3^) and As (6 ng m^-3^) concentration with 4.72% and 3.92%, while the lowest exceedance percentage was found for Ni (> 20 ng m^-3^), representing 0.79%.

### Temporal distribution of pollutants

#### Monthly and annual pollutant distribution

**Figure 1:**
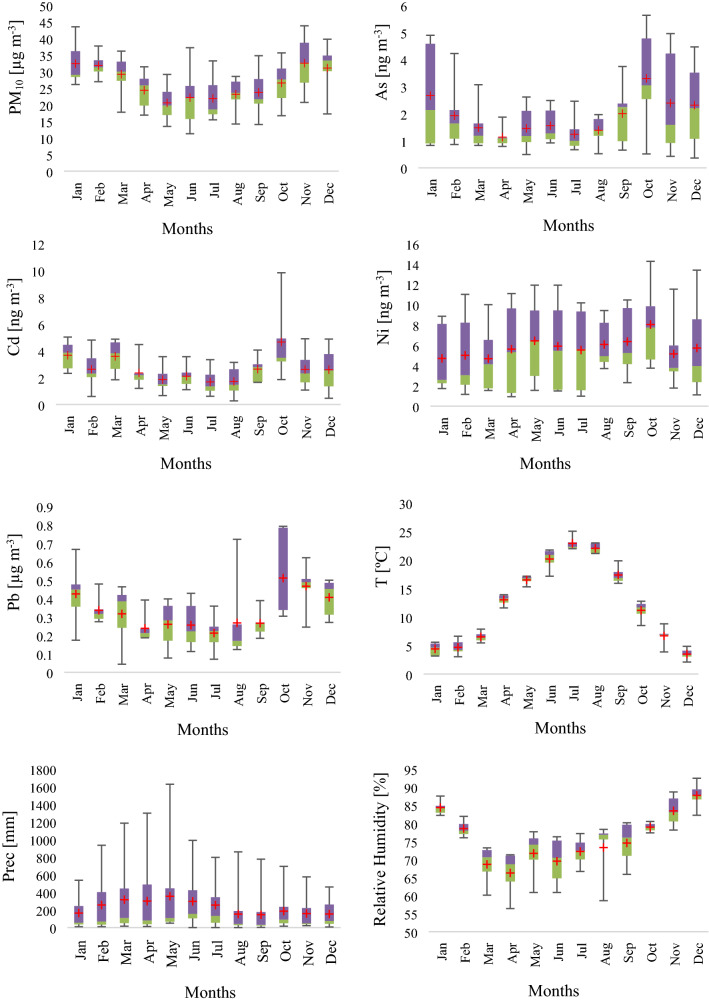
Box-plot analysis of multiannual monthly variations of pollutants and meteorological parameters. The lower (green) and upper (purple) limits represent the first (25P) and third (75P) quartiles, means are represented by red crosses, and the ends of the whiskers represent the minimum and the maximum values. The figures were prepared using the Microsoft Excel program.

The multiannual monthly variation in PM_10_ and trace elements concentrations in Copșa Mică are presented in Fig. 1. According to the results, for all studied elements (PM_10_, As, Cd, Pb) except Ni, the minimum concentration was found in the warm period, meanwhile the maximum concentration in the cold period.

**Figure 2:**
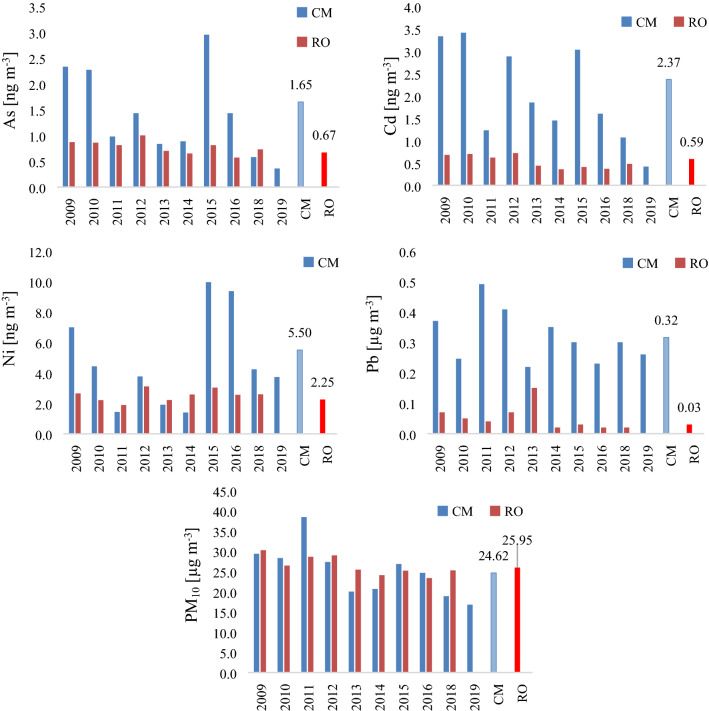
Annual variations in trace element concentrations and particulate matter. The figures were prepared using the Microsoft Excel program.

The seasonal variation of the pollutants is strongly related to different factors, mainly to the meteorological condition and emission sources, therefore the higher PM_10_ concentrations in the winter period can be explained with the domestic heating and the presence of unfavorable meteorological conditions such as thermal inversion, fog and low boundary layer height^36,37^. On the other hand, the elevated Ni concentration in summer is attributable to increased industrial production and traffic intensity^38^. The temperature and relative humidity show a negative correlation, and the highest precipitation quantity was measured in spring. Furthermore, a decreasing trend was observed during the studied period in case of all pollutants compared to the first reference year (2009). The decreasing percentage of the studied pollutants was 16.02% for PM_10_, 44.11% for As, 43.48% for Cd, 36.07% for Ni and 15.80% for Pb (Fig. 2.). One of the main reasons of decreasing air pollution level in Romania and Copșa Mică as well is, that in 2007 the country joined the European Union and became a full member state, hence stricter Environmental Protection Regulations were implemented in order to address the industrial pollution issues. During this period a modernization process has also taken place and part of the industry has been closed.

Where: *CM* and *RO* represent the multiannual average concentrations in Copșa Mică and Romanian. Compared to the multiannual country average, the Pb concentration in Copșa Mică was 10.52 times higher than the country average; even though all countries worldwide have phased out using leaded petrol by 2012, in Copșa Mică the Pb concentration determined from the PM_10_ was very high. The Pb pollution during the last decades caused by Sometra (specialized in lead, zinc and other nonferrous metals) still has a visible effect even nowadays. Since via soil resuspension, very high Pb concentrations were measured from PM_10_ samples in the studied area^39^. Moreover, similar to Pb the Cd concentration was 4.01 times higher compared to the country average, followed by the As and Ni with 2.46 and 2.44 ratio, respectively. However, the PM_10_ concentration did not differ significantly from the national average, was only 1.054 times higher than the average.

Analyzing the data on an annual breakdown, it can be seen that the highest pollutant concentration was detected in 2015. According to the 2015 annual report, we found that during this period massive rehabilitation works were carried out in different sectors including the water-sewerage-, natural gas network, and local streets/roads repairs as well^13^. Thanks to these activities dust emissions was very high, thus the particulate matter concentration in the air was sharply growing, which was the main cause of high PM_10_ levels recorded in 2015, hence the Ni concentration was also increasing. Besides the fact the precipitation amounts were considerable in 2015, they had a torrential character, after which they were accompanied by periods with persistent anticyclonic systems that induced an accentuated static stability, causing frequent periods of thermal inversion. Therefore, the washout effect on trace elements concentrations was not as significant as expected. During thermal inversions, the colder air layers were blocked under the hot air, thus preventing the formation of convection currents (ascending) and blocking the emitted noxious substances, which favors the horizontal distribution and accumulation of pollutants, especially under stable condition (no vertical mixing, air stagnation).

In order to decipher the level of pollution in Copșa Mică and the relationship with different locations worldwide, the multiannual trace element concentration measured in the study site was compared with those reported in different regions of the world (Table 2).

**Table 2:** Trace elements concentration from PM_10_ (ng m^-3^) in different regions around the world.

	As	Cd	Ni	Pb	Period	Reference
	(ng m^-3^)					
Romania	0.67	0.59	2.25	30	2009–2018	Bodor et al. under press
Romania, Copșa Mică	1.65	2.37	5.5	320	2009–2019	This study
India, Dhanbad	8.9	6.6	29.3	85.2	Sep 2014-Feb 2015	^40^
Korea, Taejon	6.75	3.28	38.27	238	1997–1999	^17^
Greece, Athens	5.68	2.8	12.48	47.85	2001–2002	^18^
USA, Appalachia	0.84	0.18	8.67	3.61	Aug-08	^41^
Taiwan, Changhua County	3.39	0.7	9.84	21.2	2013–2014	^42^
Spain, Escuelas Aguirre	1.56	0.32	2.29	13.14	Oct-Nov 2010	^43^

The comparative results revealed that the measured As concentration in Copșa Mică (1.65 ng m^-3^) was higher than what was observed in Appalachia (USA) and Spain. The Cd (2.37 ng m^-3^) concentration was the second highest after India, and the Ni concentration (5.5 ng m^-3^) was higher than what was reported in Spain. Moreover, the Pb concentration was by far the highest in Copșa Mică (320 ng m^-3^).

#### Correlation analysis

**Figure 3:**
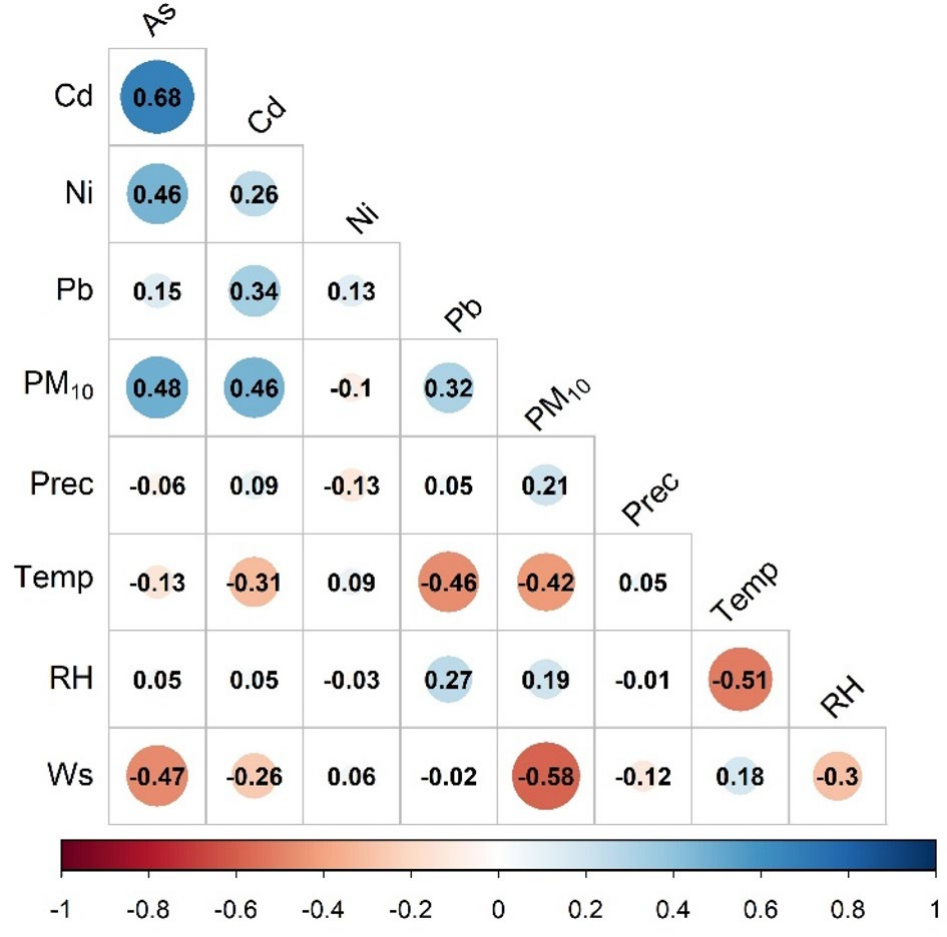
Spearman correlation coefficient matrix. The figure was prepared using R (3.6.2) statistical program.

Spearman correlation analysis was carried out between the pollutant concentration (PM_10_, As, Cd, Ni, Pb) and meteorological parameters (Prec, Temp, RH, Wind speed), using the monthly average concentrations to identify the common sources^44^. The correlation coefficients between two studied parameters were considered significant if the P < 0.05 and r ≥  + 0.27, and r ≤  − 0.27 (Fig. 3).

As expected, the results show that the most significant correlation was between As-Ni and As-Cd (r = 0.46, r = 0.68). Furthermore, significant positive correlation was found between PM_10_ and As, Cd, Pb (r = 0.48, r = 0.46, r = 0.32) and between Pb and Cd (r = 0.34) as well. The Spearman correlation shows the extent to which the magnitude of one variable determines the magnitude of the other variable, as well as the direction and strength of the relationship, hence from the significant positive correlation we can conclude whether the two variables are related or not. The correlation level of Cd-Ni, and Ni-Pb was lower than the significant level, indicating that sources of these elements are different and more diverse. The correlation matrix also indicates that significant negative correlation was between the temperature and the Cd, Pb and PM_10_ concentrations (r =  − 0.31, r =  − 0.46, r =  − 0.42), which could be attributed to the thermal inversion. Due to the fact that the days with precipitation and no precipitation were not analyzed separately, the negative correlation between the precipitation and As, Cd and Pb was not significant. The wind speed also showed a significant negative correlation with the As and Pb (r =  − 0.47, r =  − 0.58).

#### Cluster and principal component analysis

**Figure 4:**
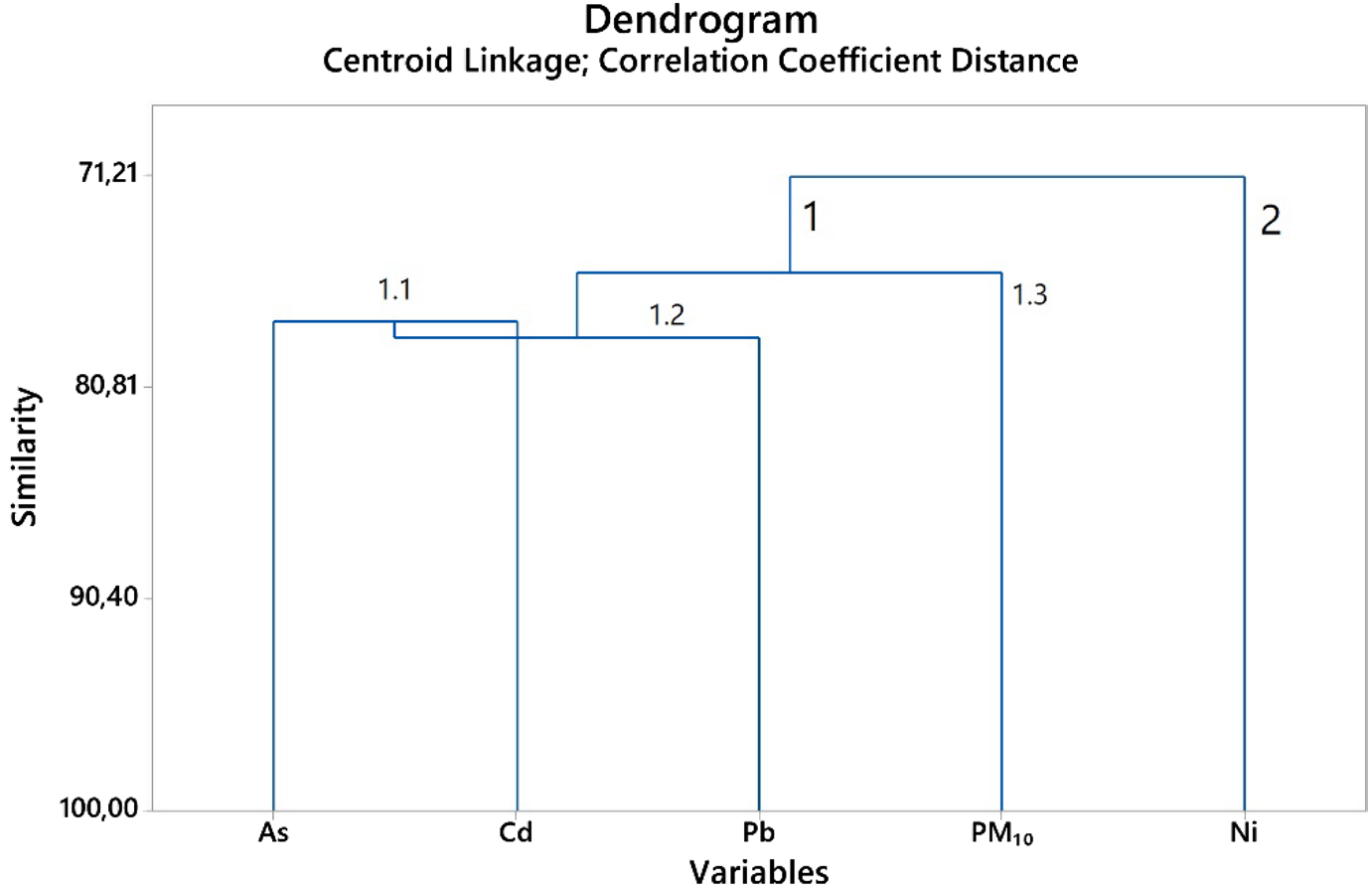
Cluster analysis of trace elements and PM_10_. The dendrogram was prepared using Minitab 17.3.1 statistical software.

Using the monthly mean concentration, hierarchical cluster analysis was performed for trace elements (As, Cd, Ni, Pb) and PM_10_ to evaluate the potential contributing sources of heavy metals. According to the hierarchical cluster analyses, the variables were distributed in two different clusters (Fig. 4.). The HCA also revealed that As and Cd belong to 1.1 sub-cluster, which means that they are coming from different sources and industrial emissions^45^. The Pb and PM_10_ form a separate sub-cluster, 1.2. and 1.3, respectively, meaning a different source, while Ni is in cluster 2, which means that can derive from motor vehicle exhaust^46^*.*

In order to identify the origin as well as the common sources of heavy metals from the PM_10_ samples, Principal Component Analysis was carried out. In the variable statement we include the first two principal components. The adequacy of the Kaiser–Meyer–Olkin (KMO) measure of sampling was 0.67, followed by the execution of the PCA, meanings that the tested samples show medium adequacy.

**Figure 5:**
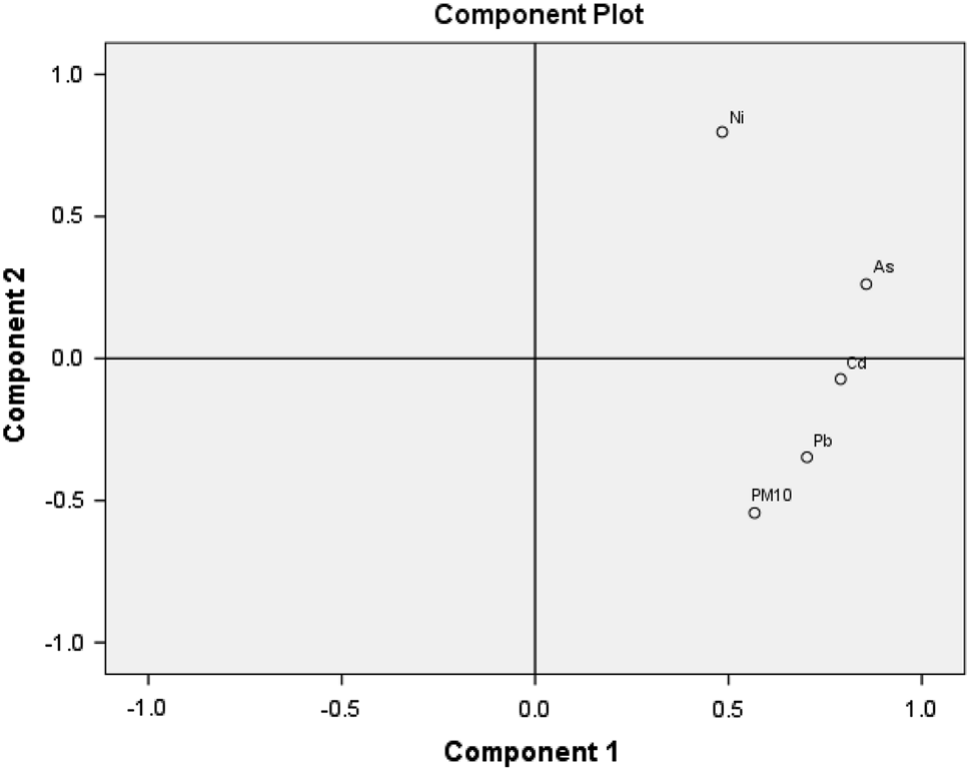
Principal Component Analysis Score plot. The figure was prepared using IBM SPSS statistics 22.

According to the results, two components were extracted from the component matrix, accounting for 70.66% of the overall variance (Table 3, Fig. 5). Factor 1 contains As, Cd, Pb, PM_10_ and represents 48.15% of the total variance, while factor two was represented by Ni.

**Table 3:** Extraction method: Principal Component Analysis.

Variable	Factor 1	Factor 2
As	0.856	0.262
Cd	0.790	− 0.072
Ni	0.484	0.797
Pb	0.703	− 0.347
PM_10_	0.567	− 0.544
Eigen value	2.408	1.126
% variance	48.15	22.51
Cumulative % variance	48.15	70.66

The most important sources of As and Cd, are related to the coal-burning, diesel fuel, lubricating oil and tire wear^47^ while the most important Pb sources are related to soil dust^48^. The origin of Ni represents factor 2 (0.797), which is due to oil burning^49^. According to the annual reports, regarding the state of the environment, the main source of county-level Pb emission is the metallurgical industry—92%—including the lead production industry from Copșa Mică (SOMETRA), the industry of other mineral products (brick), followed by domestic and institutional heating and traffic^50^. On the other hand, the highest share in Ni emission, at county-level, belongs to the industry of other mineral products, such as (brick production)—79%, followed by commercial and institutional heating, domestic heating, road transport, equipment and mobile machinery used in industries, constructions and agriculture as well.

### Health risk assessment of toxic metals in PM_10_

#### Non-cancer risk and cancer risk assessment

For the three studied exposure pathways (ingestion, inhalation, dermal absorption), the calculated HQ and CR values are presented in Table 4. According to the HQ results, the largest amount of trace elements is generally absorbed by the body through inhalation, while via ingestion and dermal absorption, for both adults and children, the HQ values were lower than the safe limit. Duo to the fact that the data for inhalation exposure route were not available, the HQ values for Pb were not calculated. Through the inhalation pathway, the HQ values were higher than the safe limit for both adults and children (= 1). The highest non-carcinogenic risk was detected for Ni (4.52), in case of both groups adults and children, while the non-carcinogenic risk value for Cd and As was of 3.89 and 1.81, respectively. However, by taking into consideration the sum of the three trace elements (HI) the intensification parameter was 1.02E + 01, showing non-carcinogen health risk by inhaling a mixture of trace elements.

**Table 4:** Hazard Quotient (HQ) and Cancer risk (CR) from trace elements in PM_10_ via ingestion, inhalation, and dermal contact for children and adults. HI*—Hazard Index.

		Ingestion		Inhalation		Dermal	
		Children	Adults	Children	Adults	Children	Adults
HQ	As	5.02E−05	5.38E−06	1.81E + 00	1.81E + 00	4.97E−06	9.04E−07
	Cd	2.16E−05	2.32E−06	3.89E + 00	3.89E + 00	2.85E−06	5.19E−07
	Ni	4.57E−06	4.90E−07	4.52E + 00	4.52E + 00	3.77E−06	6.85E−07
	Pb	8.24E−04	8.83E−05	–	–	2.72E−05	4.94E−06
	HI	9.00E−04	9.65E−05	1.02E + 01	1.02E + 01	3.88E−05	7.05E−06
CR	As	1.94E−09	8.30E−10	9.99E−06	4.00E−05	4.46E−08	6.55E−03
	Cd	1.13E−08	4.85E−09	6.01E−06	2.40E−05	8.68E−09	1.28E−03
	Ni	7.32E−09	3.14E−09	2.02E−06	8.07E−06	5.62E−08	8.26E−03
	Pb	6.92E−08	2.97E−08	5.34E−06	2.14E−05	5.31E−07	7.81E−02
	HI*	8.98E−08	3.85E−08	2.34E−05	9.34E−05	6.41E−07	9.42E−02

The results revealed that the carcinogen risk via inhalation for adults in the case of As, Cd and Pb was higher than the acceptable limit (1E−06). The total cancer risk for adults and children via inhalation was observed to be 9.34E−05 and 2.34E−05, respectively. There is a significant difference between children and adult cancer risk due to their activity and exposure time differences^51^.

Via dermal absorption and inhalation the cancer risk found was higher than the safety limit (1E−06)*;* on the other hand, adults had a higher probability of experiencing carcinogenic risk compared to the children. According to the calculations, in the case of As, adults show cancer risk via dermal absorption (6.55E−03) and inhalation (4.00E−05) while the Cd cancer risk for adults was 1.28E−03 and 2.40E−05, respectively. Via dermal absorption and inhalation, the sum of all the elements under consideration was 9.42E−02 and 9.34E−05 for adults, showing cumulative cancer risk, if the exposure is to a mixture of elements. The reason why the dermal absorption is so high may be related to the big skin contact surface. Furthermore, the cancer risk via ingestion for children and adults was below the minimum acceptable level (1E-06) for all trace elements and cumulative values as well, thus presenting a negligible carcinogen risk.

## Conclusions

The present study focused on the temporal distribution of trace elements originated from the PM_10_ in Copșa Mică, and to assess the human health effects. During the studied period (2009–2019), the temporal distribution of PM_10_ , As, Cd and Pb concentrations variation was significant, the minimum value was recorded in the warm season and the maximum concentration in the cold period. The multiannual mean concentration of PM_10_ was 24.62 µg m^-3^ exceeding the EU Air Quality admissible concentration (20.00 µg m^-3^) by 23.1%. Pb was found as the dominant metal, followed by the Ni, Cd and As. The Pb concentration was higher than the acceptable annual level in over one-fifth of the total studied days. Although more than one decade ago (2007) the Sometra Industrial company closed most of the activities, the sign of past pollution is still present and can be demonstrated even nowadays, since the trace elements in Copșa Mică such as As, Cd, Ni and Pb, are 2.46, − 4.01, 2.44, 10.52 times higher than the country average. Each element analyzed in this study could present non-carcinogenic risk via inhalation while taking together (multiple elements) a significant carcinogenic risk was revealed on adults. Based on the health risk assessment calculation, the highest Hazard Quotient was found via inhalation while exposed to mixture of trace elements, the summarized Hazard Index showed an increased level. Via dermal absorption and inhalation, a potential carcinogenic risk, exceeding the carcinogen acceptable level, was detected indicating an elevated risk of cancer to the inhabitants (adults) in the studied area. In this context, Romania needs to improve its environmental protection measures and procedures to remediate the heavily polluted industrial regions.

## References

[1] Xing YF, Xu YH, Shi MH, Lian YX (2016). The impact of PM_2.5_ on the human respiratory system. J. Thorac. Dis..

[2] Azam AG, Zanjani BR, Mood MB (2016). Effects of air pollution on human health and practical measures for prevention in Iran. J. Res. Med. Sci..

[3] Adar SD, Filigrana PA, Clements N, Peel JL (2014). Ambient coarse particulate matter and human health: a systematic review and meta-analysis. Curr. Environ. Heal. Rep..

[4] Du Y (2013). Health risk assessment of heavy metals in road dusts in urban parks of Beijing, China. Procedia Environ. Sci..

[5] Xia L, Gao Y (2010). Characterization of trace elements in PM_2.5_ aerosols in the vicinity of highways in northeast New Jersey in the U.S. east coast. Atmos. Pollut. Res..

[6] Dunea D, Iordache S, Radulescu C, Pohoata A, Dulama ID (2016). A multidimensional approach to the influence of wind on the variations of particulate matter and associated heavy metals in Ploiesti city, Romania. Rom. J. Phys..

[7] WHO. Air quality guidelines for Europe. (2019).

[8] Fomba KW (2015). Trace metal characterization of aerosol particles and cloud water during HCCT 2010. Atmos. Chem. Phys..

[9] Tian HZ (2013). A review of key hazardous trace elements in chinese coals: abundance, occurrence, behavior during coal combustion and their environmental impacts. Energy Fuels.

[10] Dai Q (2015). Characterization and source identification of heavy metals in ambient PM_10_ and PM_2.5_ in an integrated iron and steel industry zone compared with a background site. Aerosol Air Qual. Res..

[11] Song X (2015). Trace elements pollution and toxicity of airborne PM_10_ in a coal industrial city. Atmos. Pollut. Res..

[12] https://alexharford.uk/photos/romania-copsa-mica-most-polluted-town-europe.

[13] Environmental Protection Agency, S. Annual report on the state of the environment in Sibiu County. 0–224 (2015).

[14] Keresztesi, Á., Nita, I., Birsan, M., Bodor, Z. & Szép, R. The risk of cross-border pollution and the influence of regional climate on the rainwater chemistry in the Southern Carpathians, Romania. *Environ. Sci. Pollut. Reserach* (2020). 10.1007/s11356-019-07478-9 PMC7089915 31916162

[15] Keresztesi Á (2020). Assessing the variations in the chemical composition of rainwater and air masses using the zonal and meridional index. Atmos. Res..

[16] Landis MS, Norris GA, Williams RW, Weinstein JP (2001). Personal exposures to PM_2.5_ mass and trace elements in Balyimore, MD, USA. Atmos. Environ..

[17] Kim K, Lee J, Jang M (2002). Metals in airborne particulate matter from the first and second industrial complex area of Taejon city, Korea. Environ. Pollut..

[18] Manalis N (2005). Toxic metal content of particulate matter (PM_10_), within the Greater Area of Athens. Chemosphere.

[19] Mazzei, F. *et al.* Elemental composition and source apportionment of particulate matter near a steel plant in Genoa (Italy). **249**, 548–551 (2006).

[20] Prodi F (2009). Aerosol fine fraction in the Venice Lagoon: particle composition and sources. Atmos. Res..

[21] Amodio M (2013). Short communication a monitoring strategy to assess the fugitive emission from a steel plant. Atmos. Environ..

[22] Cetin B, Yatkin S, Bayram A, Odabasi M (2007). Ambient concentrations and source apportionment of PCBs and trace elements around an industrial area in Izmir, Turkey. Chemosphere.

[23] Querol X (2007). Source origin of trace elements in PM from regional background, urban and industrial sites of Spain. Atmos. Environ..

[24] Zhang Y (2009). Source apportionment of lead-containing aerosol particles in Shanghai using single particle mass spectrometry. Chemosphere.

[25] Tian, H. Z. *et al.* Trend and characteristics of atmospheric emissions of Hg, As, and Se from coal combustion in China, 1980–2007. *Atmospheric Chemistry and Physics Discussions* vol. 10 (2010).

[26] Jiun-horng T (2007). Chemical constituents in particulate emissions from an integrated iron and steel facility. J. Hazard. Mater..

[27] Pastuszka JS, Kozlowska WR, Zajusz-Zubek E (2010). Characterization of PM10 and PM25 and associated heavy metals at the crossroads and urban background site in Zabrze, Upper Silesia, Poland, during the smog episodes. Env. Monit. Assess.

[28] Hleis D, Fernández-olmo I, Ledoux F, Kfoury A, Courcot L (2013). Chemical profile identification of fugitive and confined particle emissions from an integrated iron and steelmaking plant. J. Hazard. Mater..

[29] Mohiuddin K, Strezov V, Nelson PF, Stelcer E, Evans T (2014). Mass and elemental distributions of atmospheric particles nearby blast furnace and electric arc furnace operated industrial areas in Australia. Sci. Total Environ..

[30] Taiwo AM (2014). Receptor modelling of airborne particulate matter in the vicinity of a major steelworks site. Sci. Total Environ..

[31] Proorocu M, Odagiu A, Oroian IG, Ciuiu G, Dan V (2014). Particulate matter status in Romanian urban areas: PM_10_ pollution levels in Bucharest. Environ. Eng. Manag. J..

[32] Lăcătuș R, Anca-Rovena L (2010). Evolution of heavy metals pollution from Copsa Mica. Sci. Pap. UASVM Bucharest.

[33] Bartha S, Taut I, Goji G, Vlad IA, Florin D (2020). Heavy metal content in polyfloral honey and potential health risk. A case study of Copșa Mică, Romania. Int. J. Environ. Res. Public Heal..

[34] Williamson DF, Parker RA, Kendrick JS (1989). The box plot: a simple visual method to interpret data. Ann. Int. Med..

[35] EPA (2004). Risk assessment guidance for superfund, volume I human health evaluation manual (Part A). U.S EPA.

[36] Szép R, Mateescu E, Nechifor C, Keresztesi Á (2017). Chemical characteristics and source analysis on ionic composition of rainwater collected in the Carpathians “Cold Pole”, Ciuc basin, Eastern Carpathians, Romania. Environ. Sci. Pollut. Res..

[37] Szép R (2019). Influence of peat fires on the rainwater chemistry in intra-mountain basins with specific atmospheric circulations (Eastern Carpathians, Romania). Sci. Total Environ..

[38] EMEP/EEA. Air pollutant emission inventory guidebook. *Society* 1–34 (2019).

[39] Young TM, Heerman DA, Sirin G, Ashbaugh LL (2002). Resuspension of soil as a source of airborne lead near industrial facilities and highways. Environ. Sci. Technol..

[40] Jena S, Singh G (2017). Human health risk assessment of airborne trace elements in Dhanbad, India. Atmos. Pollut. Res..

[41] Aneja VP, Isherwood A, Morgan P (2012). Characterization of particulate matter (PM_10_) related to surface coal mining operations in Appalachia. Atmos. Environ..

[42] Hsu CY (2016). Elemental characterization and source apportionment of PM_10_ and PM_2.5_ in the western coastal area of central Taiwan. Sci. Total Environ..

[43] Moreno T (2013). Daily and hourly sourcing of metallic and mineral dust in urban air contaminated by traffic and coal-burning emissions. Atmos. Environ..

[44] Javed W, Wexler AS, Murtaza G, Ahmad HR, Basra SMA (2015). Spatial, temporal and size distribution of particulate matter and its chemical constituents in Faisalabad, Pakistan. Atmosfera.

[45] Mansha M, Ghauri B, Rahman S, Amman A (2012). Characterization and source apportionment of ambient air particulate matter (PM_2.5_) in Karachi. Sci. Total Environ..

[46] Wang J, Hu Z, Chen Y, Chen Z, Xu S (2013). Contamination characteristics and possible sources of PM_10_ and PM_2.5_ in different functional areas of Shanghai, China. Atmos. Environ..

[47] Foti L (2017). Trace element concentrations along a gradient of urban pressure in forest and lawn soils of the Paris region (France). Sci. Total Environ..

[48] Zhang, Y. *et al.* Metals compositions of indoor PM2.5, health risk assessment, and birth outcomes in Lanzhou, China. *Environ. Monit. Assess.* **188**, (2016). 10.1007/s10661-016-5319-y 27147238

[49] Fang W, Delapp RC, Kosson DS, van der Sloot HA, Liu J (2017). Release of heavy metals during long-term land application of sewage sludge compost: Percolation leaching tests with repeated additions of compost. Chemosphere.

[50] Environmental Protection Agency, S. *Annual report on the state of the environment for Sibiu county*. (2019).

[51] Khairy MA, Barakat AO, Mostafa AR, Wade TL (2011). Multielement determination by flame atomic absorption of road dust samples in Delta Region, Egypt. Microchem. J..

