# Author Correction: Human health impact assessment and temporal distribution of trace elements in Copșa Mică- Romania

**DOI:** 10.1038/s41598-021-93460-w

**Published:** 2021-07-08

**Authors:** Katalin Bodor, Zsolt Bodor, Alexandru Szép, Róbert Szép

**Affiliations:** 1 Department of Bioengineering, Socio-Human Sciences and Engineering, Faculty of Economics, Sapientia Hungarian University of Transylvania, Libertăţii Sq.1, 530104 Miercurea Ciuc, Romania; 2 Doctoral School of Chemistry, Faculty of Natural Sciences, University of Pécs, Ifjúság 6, Pécs, 7624 Hungary; 3 Institute for Research and Development for Hunting and Mountain Resources, Str. Progresului 35B, 530240 Miercurea Ciuc, Romania; 4 Department of Food Engineering, Faculty of Economics, Socio-Human Sciences and Engineering, Sapientia Hungarian University of Transylvania, Libertăţii Sq.1, 530104 Miercurea Ciuc, Romania

Correction to: *Scientific Reports* 10.1038/s41598-021-86488-5, published online 29 March 2021

In the original version of this Article, Alexandru Szép was incorrectly affiliated with ‘Department of Bioengineering, Socio-Human Sciences and Engineering, Faculty of Economics, Sapientia Hungarian University of Transylvania, Libertăţii Sq.1, 530104, Miercurea Ciuc, Romania’. The correct affiliation is listed below.

Department of Food Engineering, Faculty of Economics, Socio-Human Sciences and Engineering, Sapientia Hungarian University of Transylvania, Libertăţii Sq.1, 530104 Miercurea Ciuc, Romania.

The original Article has been corrected.

